# Succession of Ground-Dwelling Beetle Assemblages After Fire in Three Habitat Types in the Andean Forest of NW Patagonia, Argentina

**DOI:** 10.1673/031.010.3701

**Published:** 2010-04-17

**Authors:** Yamila Sasal, Estela Raffaele, Alejandro G. Farji-Brener

**Affiliations:** Laboratorio Ecotono. INIBIOMA. CONICET - Universidad Nacional del Comahue, S. C. de Bariloche, Río Negro, Argentina

**Keywords:** Coleoptera, *Austrocedrus chilensis*, *Nothofagus dombeyi*, shrubland, post-fire succession

## Abstract

Wildfires are one of the major disturbances in the dynamics of forests and shrublands. However, little is known about their effects on insect communities that contribute to faunal biodiversity and play key roles in the ecosystem's dynamics. An intense and widespread fire occurred in 1999 in the Nahuel Huapi National Park in the Andean forest in northern Patagonia, Argentina. This fire affected adjacent, but different, habitat types. After the fire, beetle abundance, species richness and assemblage composition were compared among three habitats that were structurally different before the fire. These habitats were: 1) evergreen forest dominated by *Nothofagus dombeyi* (Mirb.) Oerst. (Fagales: Nothofagaceae), 2) a mixed forest of the evergreen conifer *Austrocedrus chilensis* (D. Don) Pic. Serm. and Bizzarri (Pinales: Cupressaceae) and *N. dombeyi* and 3) a shrubland with a diverse community of shrub species. The relationship between beetle diversity and vegetation structure was investigated over three consecutive years. Ground beetles were collected by pitfall traps, and plant species richness, vegetation cover, and height were measured. Beetle communities varied more over years between habitats during the early regeneration after fire. There was a shift in beetle assemblage composition with time after the fire in all habitat types, probably due to similar colonization rates and microclimatic conditions. Therefore, beetle succession was more influenced by recolonization and survivorship, accompanied by climatic conditions and recovery rate of plant communities over time, than it was influenced by pre-fire habitat conditions. These results suggest that in NW Patagonia, wildfire can have a substantial, short-term impact on beetle abundance and species composition. The pre-fire conditions of each habitat type determined the structure of post-fire communities of plants but not beetle assemblages. Wildfires produce simplification and homogenization of habitat types, and this was reflected by beetle diversity.

## Introduction

Fire is one of the most common disturbances that can cause dramatic changes in species diversity (Whelan 1995). Its effects depend on spatial heterogeneity, vegetation types, abiotic conditions, and human activities (Veblen et al. 1999; Mermoz et al. 2005). The ecological impact of fires depends on their frequency, intensity, and extent, but always causes simplification of the burned habitat (Bulan and Barrett 1971). Habitats that differ in vegetation structure may also suffer this simplification in different ways. Intense fires reduce the litter layer to mineral ash, and they kill all the above-ground vegetation. This produces changes in microclimatic conditions by increasing temperature and reducing humidity above ground (Bond and van Wilgen 1996; Paritsis et al. 2006) and below ground (Alauzis et al. 2004). This initial habitat simplification leads to high similarity among post-fire habitats in sites that differed structurally before fire. However, in different pre-fire habitat types, surviving organisms dictate much of the initial successional pattern and influence the competitive environment encountered by colonizers (Turner et al. 1998).

In northwestern Patagonia, wildfire is a major disturbance that affects the dynamics of forests and shrublands. Fires are very frequent; it has been estimated that for the last 100 years, fire intervals have ranged from 3 to 17 years (Veblen et al. 1999). Additionally, wildfires were often of high intensity and burned large areas (Veblen et al. 1999). Qualitative historical descriptions from the 18^th^ and 19^th^ centuries, as well as historical photographs from the late 19^th^ and early 20^th^ centuries, indicate that extensive, severe fires occurred in all the woodland and forest types (Veblen et al. 2003). The widespread occurrence of fires in this region in the late 1990s provided a rare opportunity to examine the effects of large wildfires on different communities. Numerous studies have documented post-fire vegetation changes in the Andean forest (Veblen and Lorenz 1987, 1988; Gobbi et al. 1995; Raffaele and Veblen 1998, 2001; Ghermandi et al. 2004) and fire history (Kitzberger et al. 1997; Veblen et al. 1999). However, few studies have addressed the effects of fire on insects and other arthropods, such as beetles, that contribute substantially to faunal biodiversity and play key roles in the ecosystem's dynamic (Farji-Brener et al. 2002; Sackmann and Farji-Brener 2006). Moreover, in this region, beetles are poorly known, are highly diverse in species number and trophic functions, and present high endemism (Morrone and Roig-Juñent 1995). Beetles have also proven to be useful bioindicators for environment monitoring and assessment, with their high diversity and sensitivity to environmental conditions providing a fine-grained view of ecological change (Orgeas and Andersen 2001). Coleoptera are known to respond to factors such as vegetation complexity and microclimate and to conditions in the soil and litter layers (Niemelä et al. 1993; Baker 2006), and these factors are likely to vary in post-fire succession. In addition, beetles are good colonizers, as many species can fly and others are active foragers that become abundant in post-fire sites (Borror et al. 1992; Swengel 2001). These reasons make beetle assemblage appropriate for studying the effects of fire disturbance on insect community structures in different habitat types.

In this study case the initial response of beetle diversity to a wildfire was examined. An intense and widespread fire occurred in 1999 that affected adjacent, but different, habitat types, allowing the study of changes over time in coleopteran assemblages under different pre-fire conditions. In northern Patagonia, following the burning of shrublands and xeric open forests, resprouting shrubs often dominate post-fire regeneration, while vegetative regeneration of tree species is rare. The establishment of tree species is often facilitated by already established shrubs that operate as nurse plants (Raffaele and Veblen 1998; Kitzberger et al. 2000). Shrubland communities are particularly well-adapted to regenerate by resprouting after fire (Veblen et al. 2003). This strategy allows this system to reach its pre-fire biomass and composition in a few years, giving it an extraordinary resilience to this kind of disturbance. Because different habitats often support different beetle assemblages (Lövei and Sunderland 1996), we hypothesized that the effect of habitat type, defined by pre-fire conditions, would determine distinct post-fire communities because beetle diversity is different among different habitats and years and because their successional patterns will be related to vegetation structure.

**Figure 1.**
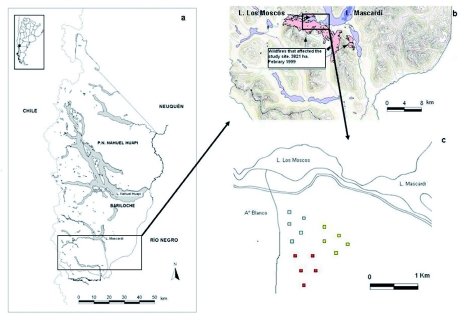
Map showing the study area a) Nahuel Huapi National Park, b) location of the wildfire and c) position of the sampling plots (blue, *Austrocedrus chilensis* forest; yellow, shrubland; red, *Nothofagus dombeyi* forest). High quality figures are available online.

The specific aims of the study were: 1) to compare beetle abundance, richness, and assemblage composition in three adjacent habitats for three years after the fire, 2) to describe the relationship between beetle diversity and vegetation structure in these habitats after the fire, and 3) to determine whether beetle abundance and richness were associated with different early post-fire mosaics of vegetation and which of the vegetation variables (e.g. plant species richness, vegetation cover, and height) could explain successional beetle assemblages of each habitat type.

## Materials and Methods

### Study area

The study was conducted in the Andean forest in northern Patagonia, Argentina, in the Nahuel Huapi National Park (40° 38′ S - 72° 42′ W; 850 m above sea level)(Figure 1a), during three growing seasons (2002, 2003, and 2004). In this region the mean minimum temperature is -2° C (July), the mean maximum is 23° C (January), and the mean annual temperature is 8° C. Precipitation is concentrated mainly in autumn and winter, and it occurs as snow, with an annual rainfall of 1700 mm (Barros et al. 1983), resulting in an asynchrony between the wet season and the growing season, and there are strong summer water deficits (Paruelo et al. 1998). At this latitude, mean precipitation decreases abruptly from about 4000 mm/yr on the western side of the Andes to less than 500 mm/yr, only 80 km to the east (De Fina 1972). As a result, forest species composition from northwestern Patagonia changes along the precipitation gradient east to west and also along the temperature gradient associated with an increase in elevation (Veblen et al. 1992). In the wetter area, the lowland rain forests are mainly dominated by the evergreen *Nothofagus dombeyi* (Mirb.) Oerst. (Fagales: Nothofagaceae). In the intermediate of the precipitation gradient, at low elevations, *N. dombeyi* forms monospecific mesic forests or mixed stands with the conifer *Austrocedrus chilensis* (D. Don) Pic. Serm. & Bizzarri (Pinales: Cupressaceae) at drier sites, and in the eastern region, this conifer forms relatively open woodlands. In the western and central areas, forest understory is typically dominated by dense and tall (> 2 m) populations of *Chusquea culeou* Desvaux (Poales: Poaceae). Tall and dense shrublands occur throughout the western to eastern precipitation gradient at sites that are not edaphically suitable for development of tall forests or that are successional communities that develop after burning of tall forests (Mermoz et al. 2005). Extensive post-fire stands of tall *Nothofagus* and/or *Austrocedrus* forests are characterized by low regeneration due to the obligate seed reproduction being able to be replaced by shrubland with vigorous post-fire resprouting (Kitzberger and Veblen 1999; Veblen et al. 2003).

In February 1999, an extensive (3821 ha) and severe wildfire affected the study site (Figure 1b) (“Administración de Parques Nacionales,” unpublished report “PN Nahuel Huapi Argentina: Incendios temporada 1998–1999”). The intense fire consumed all forest floor litter (Alauzis et al. 2004) and killed the aboveground vegetation, leaving all standing trees killed. Three adjacent habitat types were studied, each one defined by pre-fire dominating vegetation: 1) evergreen forest dominated by *N. dombeyi* (*N. dombeyi* forest) with a typically dense understory of *C. culeou,* 2) a mixed forest of the evergreen conifer *A. chilensis* (*A. chilensis* forest) and *N. dombeyi*, and 3) a shrubland (shrubland community) where a diverse community of shrub species such as *Schinus patagonicus*, *Discaria articulata* and *Lomatia hirsuta* coexist. Replication fires were not possible because no fires with the same characteristics of severity and spread (3821 ha) were found. The study area was 7 kilometres from unburned shrubland, 10 kilometres from unburned *N. dombeyi* forest, and 14 kilometres from unburned *A. chilensis* forest. It was not possible to get unburned controls because of these distances and the heterogeneity of the landscape. Also it was not possible to get prefire controls because this fire was natural and not prescribed.

### Experimental design

In the three adjacent habitat types noted above, areas were selected that were severely and homogeneously burnt in 1999. Within each habitat type, five replicate areas of 1600 m2 (40 × 40 m plots) positioned at least 100 m apart (Figure 1c) were delimited. These separation distances are within the range reported in previous studies (Rykken et al. 1997; Koivula et al. 1999), so it can be assumed to provide independent information for ecological analysis of ground-dwelling arthropods avoiding spatial autocorrelation (Moretti et al. 2004). Beetles may move up to 60 m by foot (Koivula et al. 1999), thus the scale we used in this study was appropriate for detecting habitat selection. Sampling of the beetles and vegetation was carried out during the austral summers of 2002, 2003, and 2004 because spring and summer seasons correspond to the major activity period of arthropods in this temperate region.

### Beetle sampling

Ground beetles were collected by 8 pitfall traps within each 40 × 40 m plot. Traps were placed along two transects (4 traps per transect) at 5 m intervals. Transects were placed no closer than 10 m. Each trap consisted of a 500 ml plastic cup partially filled with preservative solution (ethylene glycol, water 5:95) and buried in the soil. Pitfall traps were left open for 5 days during each January and February; this corresponded to the summer season period of thermal activity of insects (Niemelä 1990). The contents of the 8 traps of two intervals (January and February) were pooled into one sample per plot per year. Samples were sorted in the laboratory. Whenever possible beetles were identified to species, otherwise they were assigned to morphospecies. The identifications were checked and modified by appropiate specialists (see Acknowledgements). Voucher specimens are held at the Laboratory Ecotono, Universidad Nacional del Comahue, Río Negro, Argentina.

Three beetle assemblage traits were analysed: abundance, species richness and composition. Beetle abundance was expressed as the total number of individuals per plot, habitat, and year. Richness was calculated by the randomization process using EcoSim ® (Gotelli and Entsminger 2001). Rarefaction eliminated variation in species richness due to differences in sample size (e.g. number of beetles captured at each collecting station) by re-sampling a pool of individuals repeatedly, at random, on each habitat type (Gotelli and Colwell 2001).

### Vegetation sampling

To determine post-fire vegetation structure on the three habitat types over years, on each 40 × 40 m plot, 40 circular sub-plots of 80 cm radius (2 m^2^) were installed systematically on a 1 × 1 m grid. On each sub-plot the following data were recorded: 1) total number of vascular species (richness), 2) the cover of all vascular plant species using a scale from 1–100%, and 3) maximum heights of the woody species (vertical distance above the ground). Mean vegetation cover was calculated on average of 40 subplots per plot. Plant richness was not calculated by randomization because sample size was the same for all plots and sub-plots.

### Data analysis

Repeated-measures analysis of variance (ANOVA) was used to examine whether ground-active beetles responded differently over time under different habitat types (Mead 1988). Two measures of beetle response were examined: total beetle abundance and rarefied beetle species richness. The analysis included years and habitat types (*N. dombeyi* forest, *A. chilensis* forest and shrubland community) as fixed factors. Tukey tests were used for posteriori comparisons (Sokal and Rohlf 1995). Analysis of similarity (ANOSIM) with Bray-Curtis similarity index was used to determine if there were significant differences in beetle assemblages during the years since the fire (2002, 2003, and 2004) and among habitat types (*N. dombeyi* forest, *A. chilensis* forest and shrubland community). ANOSIM is a non-parametric permutation procedure applied to rank similarity matrices underlying sample ordinations (Clarke and Warwick 2001), that produces a global R-statistic, which is an absolute measure of distance between groups. An R-value approaching 1 indicates strongly distinct assemblages, whereas an R-value close to zero indicates that the assemblages are barely separable. To illustrate patterns in beetle assemblage composition in relation to habitat types and years, non-metric multidimensional scaling (NMDS) ordination with the Bray-Curtis similarity index (Clarke 1993) was used. Beetle species characteristics of the three habitat types and years were identified using the Indicator Value method (Dufrêne and Legendre 1997). This method assesses the degree to which a species fulfills the criteria of specificity (uniqueness to a particular habitat) and fidelity (frequency of occurrence). A high indicator value (IndVal, expressed as percentage) indicates that a species can be considered characteristic of a particular habitat or site. This method can derive indicators for hierarchical and non-hierarchical site classifications and is robust to differences in the numbers of sites among site groups (McGeoch and Chown 1998). Indicator values for each species were calculated based on a species abundance matrix, and Dufrêne and Legendre's (1997) random reallocation procedure of sites among site groups was used to test for the significance of IndVal measures for each species. Dufrêne and Legendre (1997) was followed in assuming a species is characteristic of a habitat if the species IndVal is > 25% and significant at p < 0.05.

Overall, to test differences in vegetation structure (plant species richness, vegetation cover and height) among habitats over time, repeated-measures ANOVA (Mead 1998) were performed including habitat types and years as fixed factors. Tukey tests were used for posteriori comparisons (Sokal and Rohlf 1995). Analysis of similarity (ANOSIM) with Bray-Curtis similarity index was used to determine if there were significant differences in vegetation assemblages between years since fire (2002, 2003, and 2004) and among habitat types (*N. dombeyi* forest, *A. chilensis* forest and shrubland community). To illustrate patterns in vegetation assemblage composition in relation to habitat types and years, non-metric multidimensional scaling (NMDS) ordination was used with the Bray-Curtis similarity index (Clarke 1993).

To describe the relationship between beetles and vegetation, correlations were performed between beetle abundance and total species richness with vegetation structure parameters (richness, cover, and height). Because different vegetation types could recover at different rates and because beetles could respond to different characteristics of these habitats, the relationship between beetles and vegetation was calculated with data of the last year, 2004. Additionally, to describe the relationship between beetle and vegetation composition, a Mantel permutation test was performed with 10,000 randomizations (Mantel 1967). The beetle and vegetation similarity matrixes were constructed using Bray-Curtis similarity measures per habitat type only for the last year, 2004 (Legendre and Legendre 1998). To explain the successional relationship between beetle assemblages with vegetation variables over years, the BIOENV procedure was used on each habitat (Clarke and Ainsworth 1993). BIOENV produces a rank-correlation coefficient among measures for the biological distance among years on each habitat (calculated by using assemblage composition data for each habitat over years) plotted against measures for environmental distance among habitats (calculated by using vegetation data for each habitat over years). The beetle and vegetation similarity matrix were constructed using Bray-Curtis similarity measures per habitat type over years (Legendre and Legendre 1998). Spearman's rank correlation coefficients (p) were then calculated for the species matrix and each of the vegetation variables (richness, vegetation cover and vegetation height per habitat type). The variable or set of variables that have the highest ρ-value are those that best explain the beetle species data (Clarke and Gorley 2001).

**Table 1.**
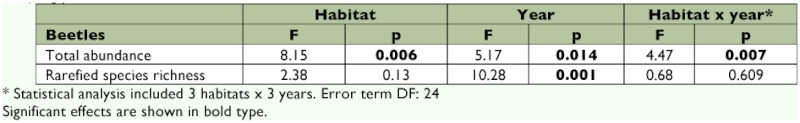
ANOVA's results for beetle total abundance and rarefied species richness at different habitat types. during 2002–2004 sampling periods.

**Table 2.**
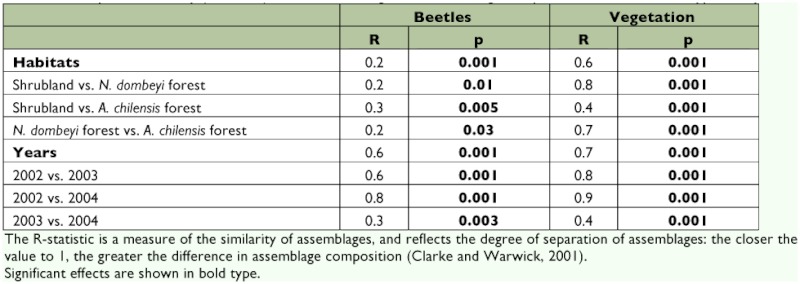
Analysis of similarity (ANOSIM) for beetle and vegetation assemblage composition between habitat types and years.

## Results

### Beetle succession

Over the three years following the wildfire, a total of 2,734 adult beetles were collected from 57 species belonging to 24 families. The presence and location of beetle species caught are shown in the Appendix. The dominant families were Carabidae with 11 species, comprising 70% of all individuals collected, followed by Staphylinidae with 7 species (17%), Coccinelidae with 6 species (1.5%), Curculionidae with 4 species (0.3%) and Leiodidae with 4 species (2.5%) (Appendix). The majority of beetles collected were predators like Carabidae, Staphylinidae and Coccinelidae, but some families were herbivorous like Curculionidae or scavengers like Leiodidae.

**Figure 2.**
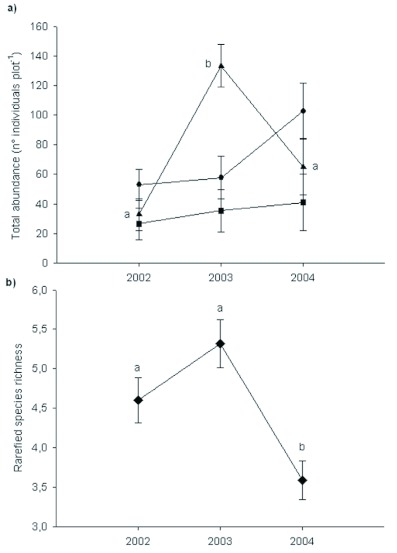
a) Total beetle abundance (mean ± SE) at different habitat types: 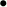 *Nothofagus dombeyi* forest, 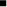 *Austrocedrus chilensis* forest, and 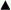 shrubland community; and b) Beetle rarefied species richness (mean ± SE) per year. Different letters above error bars denote significant differences (Tukey test, 95%). High quality figures are available online.

The total beetle abundance showed differences in habitat types over years, between habitat types and among years (Table I). In the habitat types over years, the total beetle abundance in the shrubland community was lowest in 2002, reached a maximum in 2003, and decreased to intermediate values in 2004 (Tukey, *p* = 0.004)(Figure 2a). However, in the *N. dombeyi* and *A. chilensis* forest, the abundance increased over years and the difference between years was not significant (Figure 2a).

Beetle rarefied species richness was similar between habitat types but varied over years (Table 1). On the three habitat types, species richness was intermediate in 2002, highest in 2003, and in 2004 it was significantly lower than in 2002 and 2003 (Figure 2b).

**Figure 3.**
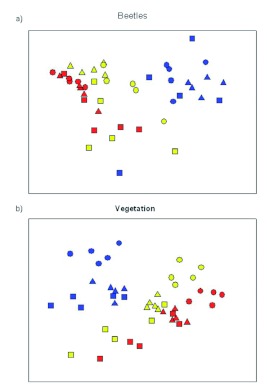
Non-metric multidimensional scaling ordination plots of beetle (stress = 0.14) and vegetation (stress = 0.17) assemblages based on habitats and years. Habitats: 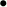 *Nothofagus dombeyi* forest, 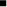 *Austrocedrus chilensis* forest, and 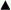 shrubland community. Years: blue, 2002; yellow, 2003; and red, 2004. For ANOSIM results, see Table 2. High quality figures are available online.

Beetle assemblage composition was similar among habitats and presented different patterns over post-fire years (Table 2). Ordination of beetle assemblages in relation to differences in habitat types and years (Figure 3a) illustrates that years is the stronger influence on beetle assemblages. Beetle assemblage presented a transition in composition from 2002 in the right, 2003 in the middle, and 2004 to the left of the figure (Figure 3a). Although there was some overlap in beetle assemblage composition in years 2003 and 2004, there was a distinct cluster for year 2002. Furthermore, beetle assemblages within years did not differ among habitat types (Figure 3a).

Indicator species analysis to identified species characteristics of habitat types was low (60%); however, the indicator value for years was higher with a maximum of 80%. For habitat types, indicator species analysis identified two species characteristic of shrubland community, one species characteristic of *N dombeyi* forest, and two species of *A. chilensis* forest (Table 4). Four species were characteristic of 2002, five species of 2003, and three species of 2004 (Table 4).

### Vegetation structure and beetle assemblages

Early post-fire vegetation succession was different between habitat types and years. Plant species richness differed between habitats and years (Table 3). The *A. chilensis* forest (47.4 ± 1.8) had more species than the shrubland community (38.7 ± 1.8; Tukey, *p* = 0.02) and the *N. dombeyi* forest (37.7 ± 1.8; Tukey, *p* = 0.005). Plant species richness was very low in 2002 (38.5 ± 1.2) compared with 2003 (42.6 ± 0.9; Tukey, *p* = 0.0002), and 2004 was similar to 2003 (42.2 ± 1.4; Tukey, *p* = 0.0005). Vegetation cover and height were similar among habitats but differed among years (Table 3). Total vegetation cover was very low in 2002 (70.5% ± 3.3) and differed from 2003 and 2004. In 2003, it increased to 101.7% ± 2.6 (Tukey, *p* = 0.0001), and in 2004, it was similar to 2003 (118.91% ± 3.32; Tukey, *p* =0.0001). On the other hand, vegetation height increased over years and differed between them (Tukey, *p* *<* 0.05). In 2002, vegetation reached 80.0 cm ± 1.9, and, in 2003, it reached 101.7 cm ± 2.6. It was 118.9 cm ± 3.3 in 2004. Plant assemblage composition differed between habitats and years (Table 2). Ordination of plant assemblages in relation to habitat types and years differences (Figure 3b) illustrates that both influence vegetation assemblages. On the top, *N. dombeyi* forest was separated from the shrubland and *A. chilensis* forest, which were overlapped at the bottom (Table 2, Figure 3b). Among years, plant species composition presented a transition from 2002 in the left, to 2003 in the middle, and 2004 to the right (Table 2, Figure 3b); however, years 2003 and 2004 overlapped (Figure 3b).

**Table 3.**
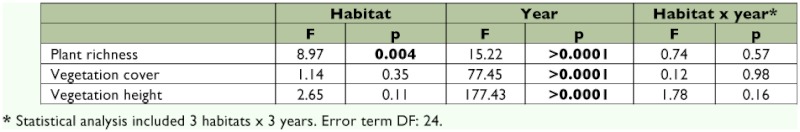
ANOVA results for plant species richness, vegetation height and cover at different habitat types, during 2002–2004.

**Table 4.**
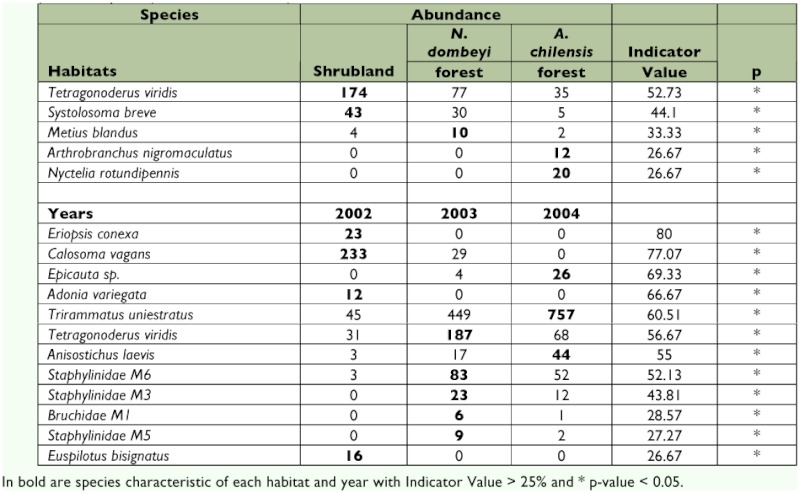
Abundance and Indicator Value (%) of beetle species for each habitat (shrubland community, *N. dombeyi* and *A. chilensis* forest) and over years (2002, 2003 and 2004).

**Figure 4.**
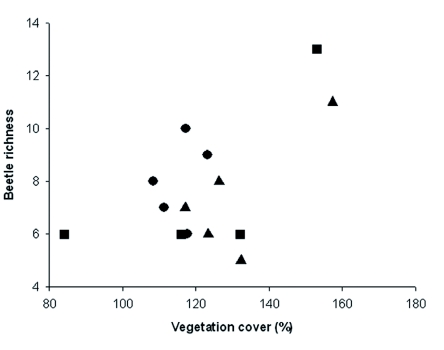
Correlation between beetle species and vegetation cover (r = 0.54, *p* = 0.038). 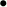 *Nothofagus dombeyi* forest, 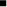 *Austrocedrus chilensis* forest, and 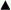 shrubland community. Only significant correlations are shown. High quality figures are available online.

Only one significant relationship was found between beetle diversity and structural vegetation characteristics (richness, height, and cover) including the three vegetation types together. Beetle richness increased with vegetation cover (r = 0.54, *p* = 0.04) (Figure 4). There was no significant relationship between beetle abundance and plant richness (r = 0.3, *p* = 0.2), vegetation cover (r = 0.2, *p* = 0.5) or vegetation height (r = 0.2, *p* = 0.5), nor between beetle abundance and vegetation richness (r = 0.03, *p* = 0.9), and vegetation height (r = 0.3, *p* = 0.3). Mantel test detected a positive association between coleopteran assemblage and vegetation assemblage for 2004 data (r = 0.48, *p* = 0.004). In addition, BIOENV analysis revealed that beetle species composition over years was best explained by vegetation cover (ρ = 0.8) in shrubland, by plant richness (ρ = 0.6) in *N. dombeyi* forest, and by vegetation cover (ρ = 0.4) in *A. chilensis* forest.

## Discussion

### Beetle succession

Although these results arise from a case study (one large fire), this research offers strong evidence over time (three consecutive years) of post-fire beetle succession in Patagonia. The assumption was that habitat pre-fire conditions would determine different post-fire beetle communities. However, the results indicated that beetle communities varied more over years than among habitats during the early regeneration after fire. Different beetle abundance patterns were found over years on each habitat type. Beetle abundance in shrubland peaked in 2003, but, beetle abundance increased over three years in *N. dombeyi* and *A. chilensis* forest. Beetles rarefied species richness for all habitats also varied among years, with a highest number of species in 2003. Despite the number of beetle species found - it was relatively low compared to previous results (Sackmann and Farji-Brener 2006) - differences were found between years. Shrubland abundance and rarefied richness both showed the highest value for 2003. This could be due to several non-exclusive causes. One possible explanation could be beetle species survival and arrival after the wildfire (Moretti et al. 2006). Some species could have benefited from recent post-fire conditions and immigrated to the burned area and, also, some species could survive wildfire, varying abundance and richness over years. Another explanation could be due to climatic variations among years and extreme climatic events (e.g. droughts). These events are known to alter life history traits such as breeding phenology (Ayres and Lombardero 2000). During the 3-yr study period, environmental conditions varied markedly (INTA Bariloche), particularly precipitation. The 2001–2002 growing season was dry (October-March: 27.16 mm/month). In contrast, the 2002–2003 growing season was extremely wet (55.33 mm/month), and the 2003–2004 growing season was the driest (22.16 mm/month). Blanch et al. (2001) showed that the number of individuals, families, and species was strongly determined by seasonal and yearly patterns of rainfall. When substantial rain fell before the time of sampling, total beetle abundance and richness increased markedly (Blanche et al. 2001). Moreover, shrubland plant communities are well adapted to regenerate by resprouting after fire (Veblen et al. 2003). This plant community could have taken advantage of this limiting resource (rain), increasing growth and indirectly influencing the ability of beetles to obtain food and shelter, as well as directly affecting microclimatic conditions necessary for their survival (Niwa and Peck 2002). Overall, these variations in precipitation could explain the pattern found in beetle abundance in shrubland and species richness over years in all habitats, which were highest in 2003.

Parallel to the results of beetle abundance and richness, differences in beetle species composition were found between years rather than between habitats. There was a shift in beetle assemblage composition with time since fire in all habitat types. The year 2002 was very different from the years 2003 and 2004, and all habitats showed similar beetle composition over the years. This successional beetle pattern occurred concurrently with the vegetation successional pattern, although vegetation also changed among habitats. As a result, beetle succession could be more influenced by recolonization and survivorship, accompanied by climatic conditions and recovery rate of plants communities over years (Bess et al. 2002) than by pre-fire habitat conditions. All habitats in 2002 supported species that may have colonized the recently burned habitat and/or species that may have survived the disturbance (Baker 2006), such as *Eriopsis conexa*, *Calosoma vagans*, *Adonia variegata* and *Euspilotus bisignatus*. These species are from the families Histeridae, Tenebrionidae and Coccinelidae, which are associated with open habitats that seemed to have benefited from habitat alteration induced by fire (Sackmann and Farji-Brener 2006). However, in 2003 and 2004, characteristic species were present in both years, but their abundance changed. In 2003, *Tetragonoderus viridis* (Carabidae) and morphospecies of Staphilinidae and Bruchidae were the characteristic species. The families Carabidae and Staphilinidae are predators, and Bruchidae are seed predators (Borror et al. 1992). Staphilinidae were associated with decaying materials and moist microsites, such as under stones and other objects on the ground. In 2004, *Trirammatus uniestratus* and *Anisostichus laevis*, both Carabidae (predators), and *Epicauta sp.* (Meloidae) (herbivorous) were the characteristic species. Assemblage composition appeared to shift rapidly 3–5 years after the fire as was previously found by Bess et al. (2002). These results suggested that wildfire in NW Patagonia can have a substantial, short-term impact on beetle abundance and species composition, but further research following the post-fire succession over time would be worthwhile.

On the other hand, vegetation diversity varied with both habitat types and years since the fire. Post-fire plant species richness and composition differed among habitat types, and there were changes in vegetation cover, height, plant richness and composition over years. *Austrucedrus chilensis* forest had the highest plant richness, probably because it was the driest (Veblen et al. 2003), and it also had the most heterogeneous habitat in terms of light and soils. This environmental variability and heterogeneity led to the establishment of more plant species. Conversely, *N. dombeyi* forest differed in species composition from shrubland and *A. chilensis* forest. Those two habitats shared species and species abundance, despite differing in species richness. Plant richness, vegetation cover, and composition increased greatly from 2002 to 2003. This plant recolonization was probably due to the climatic variations and extreme events discussed above. Overall, pre-fire conditions determined different post-fire plant communities and successions in each habitat type.

It was predicted that beetle successional patterns would be related to vegetation structure, and the results partly support this prediction because there was succession over time in plants and beetles, but vegetation communities were also different between prefire habitat types. Beetle richness increased with vegetation cover, taking into account the three habitats together in 2004. Areas with more vegetation cover could be associated with habitat availability for many arthropods (Abensperg-Traun et al. 1996; Suominen et al. 1999), whereas this relationship was similar for the three habitats and consistent with the findings of previous studies conducted in the region (Sackmann and Farji-Brener 2006). Additionally, there was a positive correlation between beetle and vegetation assemblages in 2004. Beetle and vegetation assemblage succession patterns were parallel over years but not among habitats. There were differences in vegetation assemblages among habitats. Over years, beetle assemblage was related with vegetation cover in shrubland and the *A. chilensis* forest, however, beetle assemblage was related to plant richness in the *N. dombeyi* forest.

Pre-fire conditions of each habitat type determined different post-fire communities of plants, but not of beetles. The pattern of beetle succession was more influenced by the time since the fire than by habitat conditions prefire. Wildfires produce simplification and homogenization of habitat types, and this was reflected by beetle diversity. These findings suggest that beetles might not provide effective indicators of pre-fire habitat types.
